# Rec8 Cohesin: A Structural Platform for Shaping the Meiotic Chromosomes

**DOI:** 10.3390/genes13020200

**Published:** 2022-01-22

**Authors:** Takeshi Sakuno, Yasushi Hiraoka

**Affiliations:** Graduate School of Frontier Biosciences, Osaka University, Suita 565-0871, Japan; sakuno@fbs.osaka-u.ac.jp

**Keywords:** meiosis, chromosome, cohesin, Rec8, Pds5, Wpl1, synaptonemal complex, axis-loop structure, homolog pairing, recombination

## Abstract

Meiosis is critically different from mitosis in that during meiosis, pairing and segregation of homologous chromosomes occur. During meiosis, the morphology of sister chromatids changes drastically, forming a prominent axial structure in the synaptonemal complex. The meiosis-specific cohesin complex plays a central role in the regulation of the processes required for recombination. In particular, the Rec8 subunit of the meiotic cohesin complex, which is conserved in a wide range of eukaryotes, has been analyzed for its function in modulating chromosomal architecture during the pairing and recombination of homologous chromosomes in meiosis. Here, we review the current understanding of Rec8 cohesin as a structural platform for meiotic chromosomes.

## 1. Introduction

Cohesin is a highly conserved protein complex that establishes cohesion between sister chromatids after DNA replication, and is essential for proper chromosome segregation during mitosis and meiosis [1,2]. Rec8, a meiosis-specific component of cohesin, plays a central role in the recombination of homologous chromosomes during meiosis [3,4].

Meiosis is a unique type of eukaryotic cell division that produces gametes which are essential for sexual reproduction. In meiosis, one round of DNA replication is followed by two successive rounds of chromosome segregation, thus resulting in half the number of chromosomes in the gametes. Meiosis produces sperm and eggs in humans, and abnormalities in meiosis can cause miscarriages as well as various congenital genetic disorders, such as Down syndrome [5,6].

**Figure 1:**
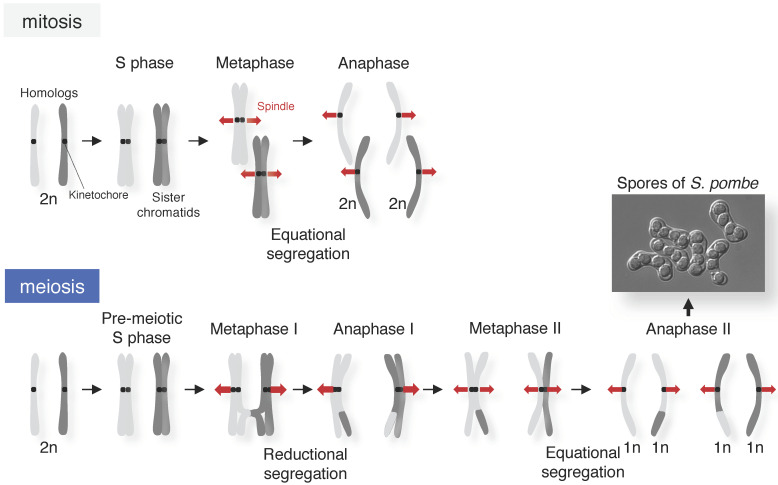
Chromosome segregation during mitosis and meiosis. Schematic diagram of chromosome segregation patterns during mitosis and meiosis. The picture shows spores of *Schizosaccharomyces pombe* produced after meiosis.

**Figure 2:**
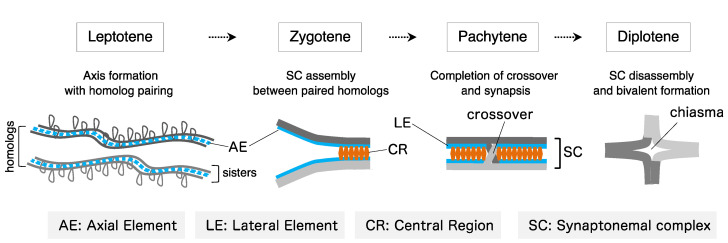
Chromosome organization during meiotic prophase. Schematic representation of chromosome organization during meiotic prophase in synaptonemal complex (SC)-forming organisms. Meiotic prophase is divided into four distinct stages: leptotene, zygotene, pachytene, and diplotene. The axial element (AE) is the precursor of the lateral element (LE) of the SC.

In the first meiotic division (meiosis I), homologous chromosomes segregate to opposite poles. This process is called reductional segregation and is essentially different from both mitosis and the second meiotic division (meiosis II), in which sister chromatids (a pair of replicated chromosomes) segregate to opposite poles (Figure 1) [3]. To correctly accomplish reductional segregation in meiosis I, homologous chromosomes need to be physically linked to each other via the chiasmata, a product of crossing over (the exchange of DNA sequences between homologous chromosomes) during the meiotic prophase. In almost all eukaryotes, homologous chromosomes are spatially separated in the nucleus during mitosis, but they become paired during the meiotic prophase, by first identifying the correct partner and then moving into physical proximity (pairing). Pairing is stabilized by the synaptonemal complex (SC), which holds homologous axes together along its entire length (synapsis) (Figure 2). The SC is composed of two lateral elements, each of which is derived from an axial element formed along a pair of sister chromatids, and the central region, which is composed of transverse filaments (i.e., Zip1 in budding yeast or SYCP1 in mice) which connect the lateral elements of each homologous chromosome (Figure 2) [7,8]. However, homologous chromosomes can pair without SCs, even in SC-forming organisms [9,10,11]. Moreover, homologous pairing also occurs in asynaptic organisms, such as the fission yeast *Schizosaccharomyces pombe*, which does not form a typical SC [12,13,14].

The Rec8-containing cohesin complex (Rec8 cohesin) is essential for the assembly of lateral elements, which are required for SC formation and homologous recombination in a wide range of eukaryotes [15,16,17,18,19,20,21,22,23,24,25]. Even in asynaptic organisms, Rec8 cohesin forms an axial structure resembling the lateral element, which is essential for homologous recombination during meiosis [26,27,28,29,30,31,32]. In addition to these canonical roles of Rec8 cohesin in recombination, recent developments in technologies, such as genome-wide chromosome conformation capture, have revealed roles for Rec8 cohesin in meiotic chromosome architecture [33,34,35,36]. This review highlights the role of Rec8 cohesin in meiosis, which is common in both SC-forming and asynaptic organisms.

## 2. Components of Cohesin Complexes

**Figure 3:**
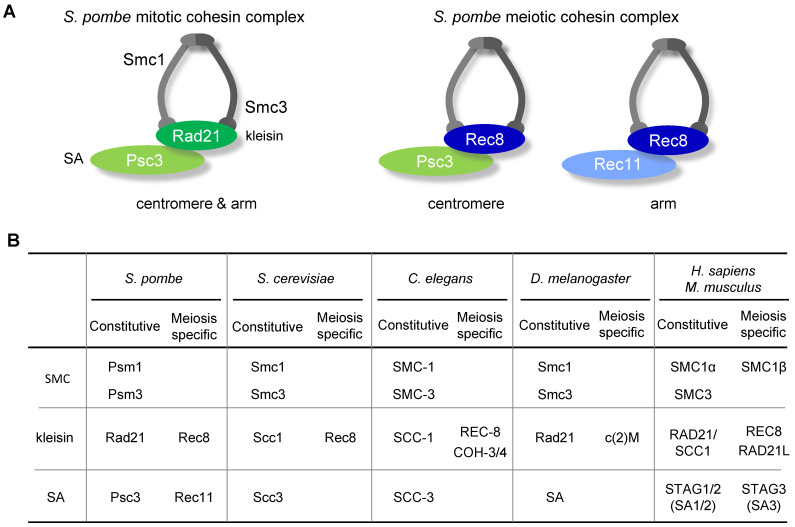
Cohesin complex in mitosis and meiosis. (**A**). A schematic diagram of cohesin complexes in *Schizosaccharomyces pombe*. During mitosis in *S. pombe*, the Rad21-Psc3 cohesin complex is distributed at the centromere and along the chromosome arm. During meiosis, the Rec8-Psc3 cohesin complex is localized only at the centromere, whereas the Rec8-Rec11 cohesin complex is distributed along the chromosome arms. (**B**). Nomenclature for the subunits of the cohesin complex in representative eukaryotic model organisms. The constitutively expressed subunits and the meiosis-specific subunits are shown.

The mitotic cohesin complex consists of four core subunits, two structural maintenance of chromosome (SMC) family proteins, Smc1 and Smc3, and two non-SMC kleisin subunits, Scc1 and Scc3 (Scc1 is also called Mcd1 in the budding yeast *Saccharomyces cerevisiae*, and Rad21 in several other organisms; Scc3 is also called SA1/2 in metazoans, and Psc3 in *S. pombe*) (Figure 3A,B) [37]. It has been proposed that the cohesin complex forms a ring structure that topologically entraps two sister chromatids together; alternatively, two cohesin rings, each entrapping one sister chromatid, are held together [38,39].

The meiotic cohesin complex uses subunits that differ from those involved in mitosis. In most species, the vast majority of Rad21 and SA1/2 subunits are replaced by meiosis-specific subunits, namely Rec8 and SA3, respectively (Figure 3B). In vertebrates, Smc1β is expressed in addition to Smc1, and a meiosis-specific kleisin, Rad21L, is also expressed [40,41,42]. In many species, these meiosis-specific subunits are required not only for sister chromatid cohesion, but also for the formation of the axial element, which serves as a scaffold for recombination reactions within the SC [8].

## 3. Roles of Meiotic Cohesin in Chromosomal Events

Homologous chromosomes must be paired and recombined during meiosis to ensure proper segregation of homologous chromosomes. Meiosis-specific subunits of cohesin play an essential role in these meiotic chromosomal events.

### 3.1. Pairing of Homologous Chromosomes

**Figure 4:**
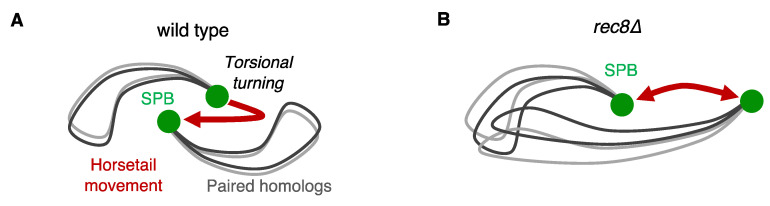
Horsetail nuclear movement in meiosis. Schematic diagrams of chromosome movement in wild-type (**A**) and *rec8∆* (**B**) cells.

A striking example of asynaptic pairing of homologous chromosomes can be observed in *S. pombe.* In *S. pombe*, pairing of homologous chromosomes depends highly on the oscillatory movement of the nucleus (so-called “horsetail” movement) with the telomeres bundled at the spindle pole body (SPB) generating a bouquet orientation of chromosomes [26,43,44,45,46,47]. In *S. pombe*, the pairing of homologous chromosomes is achieved during horsetail nuclear movement in the absence of DNA double-strand breaks (DSBs) introduced by Rec12 (Spo11 in *S. cerevisiae*). Nevertheless, pairing of homologous chromosomes requires Rec8 cohesin, indicating that meiotic cohesin plays a role in homolog pairing independent of Rec12-mediated DSB or SC formation. Torsional turning of chromosomes, which occurs when the SPB changes the direction of nuclear movement, is thought to promote the alignment of homologous chromosomes (Figure 4A) [48]. Such chromosome movements promote homologous pairing and at the same time, eliminate non-homologous interaction [48,49]. Meiosis-specific chromosome movements caused by the attachment of telomeres to the nuclear membrane also contribute to the initiation of pairing in other organisms [50,51].

Additionally, it has been shown that in *S. pombe*, noncoding RNAs and their associated factors, called *sme2* RNA-associated proteins (Smp), contribute to the DSB-independent pairing of homologous chromosomes [43,45,52]. It should be noted that non-coding RNA-mediated pairing of homologous chromosomes in *S. pombe* requires Rec8 [45,53,54,55]. In *rec8∆* meiotic cells of *S. pombe*, the oscillatory movement of SPB and bouquet orientation appear to be normal [32,54,55]. However, the bulk of the chromosomes are stuck, protruding from the leading edge of the nucleus during horsetail movement, resulting in defective pairing in *rec8∆* cells (Figure 4B) [54,55]. Therefore, during horsetail nuclear movement, compromised chromosome motion in *rec8∆* cells without torsional turning of chromosomes could lead to pairing defects. This abnormal chromosomal behavior in *rec8∆* cells might occur because the traction force that drives whole chromosomes is not well transmitted to the entire chromosome, owing to a loss of chromosome structure in the absence of Rec8 cohesin.

### 3.2. Regulation of Recombination Bias between Homologous Chromosomes and Sister Chromatids

During the repair of DSBs, which occurs spontaneously during mitosis, Scc1/Rad21 mitotic cohesin promotes damage-induced homologous recombination between sister chromatids, suppressing recombination between homologous chromosomes [56]. This is called sister bias, and is presumably caused by the spatial proximity of sister chromatids due to cohesin-dependent sister chromatid cohesion. In contrast, during meiotic prophase, DSBs introduced by Spo11 are repaired by homologous recombination, which preferentially uses homologous chromosomes and not sister chromatids as templates, a mechanism called homolog bias [57,58]. The physical proximity of homologous chromosomes via pairing or SC formation is an essential prerequisite for establishing homolog bias. In addition, studies in *S. cerevisiae* have suggested that this bias is facilitated by the cooperative function of Rec8 cohesin with components of axial elements, such as Red1, Hop1, and Mek1 [59,60]. Interestingly, in the absence of these axial components, sister bias was induced in a Rec8 cohesin-dependent manner, suggesting that Rec8 cohesin which is responsible for sister chromatid cohesion during meiosis also potentially plays a role in sister bias in Spo11-induced meiotic recombination as well as Scc1/Rad21 cohesin during mitosis [59]. However, the functions of Rec8 cohesin can be modified and regulated to promote homolog bias by several factors, including components of the axial element. The conservation of this homolog bias across eukaryotes is not apparent, but it seems to be absent in fission yeast [61].

### 3.3. Segregation of Homologous Chromosomes and Sister Chromatids

**Figure 5:**
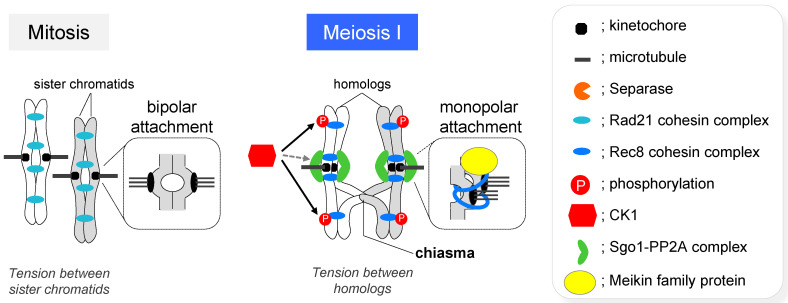
Molecular details of the regulation of chromosome segregation in meiosis I. A schematic diagram of the state of chromosomes immediately before segregation in mitosis (**left**) and meiosis I (**right**).

After physical links are established between homologous chromosomes via the chiasmata as a result of crossing over, homologous chromosomes segregate to opposite poles (reductional segregation) during meiosis I. In metaphase I, sister kinetochores behave as a single unit and establish mono-polar attachment; specifically, sister kinetochores are captured by spindle microtubules emanating from one pole (Figure 5). Because of the cooperative action of this mono-polar attachment and the chiasmata, tension is generated between homologous chromosomes; thus, spindles successfully pull homologous chromosomes toward both poles [3,15,62,63,64,65,66,67,68,69]. In *S. pombe*, it has been shown that in meiosis I, sister kinetochores are connected by the Rec8 cohesin-mediated cohesion around the centromeric region [70]. More recently, this type of Rec8 cohesin-dependent cohesion at the centromere was shown to be necessary for mono-polar meiosis I attachment in female mice [71]. The establishment of Rec8 cohesin-dependent centromeric cohesion is regulated by meiosis-specific kinetochore factors called Meikin family proteins, Moa1 in *S. pombe*, Spo13 in *S. cerevisiae*, and Meikin in mice [72,73,74,75,76,77]. The precise functions of Meikin family proteins are still unknown, but it has been reported that Meikin recruits polo-like kinases to the kinetochore, and this kinase has essential functions in establishing mono-polar attachment [72,73,74]. In *S. cerevisiae*, in addition to regulation by Spo13, mono-polar attachment is also regulated by a different set of meiosis-specific kinetochore protein complexes called monopolins, including Mam1, Csm1, Lrs4, and Hrr25 [68,69,78].

Rec8 cohesin holds homologous chromosomes together via sister chromatid cohesion, and this state is maintained in the vicinity of the crossing over site until anaphase I, resulting in retention of physical interaction of homologous chromosomes via the chiasma [3,79]. Subsequently, at the onset of anaphase I, cohesin complexes are dissociated from chromosomes by separase, a cysteine endoprotease which cleaves the kleisin subunit of cohesin, thus allowing the separation of homologous chromosomes. Importantly, although cohesin at the chromosome arms is degraded by separase in anaphase I, cohesin at the centromere is protected from degradation [15,80]. As a result, sister chromatids remain associated at the centromere through cohesion via Rec8 cohesin. Subsequently, in meiosis II, sister chromatids are evenly segregated, similar to somatic cell division, by releasing the remaining cohesion at the centromeres (Figure 5). It has been reported that the frequency of crossing over at centromeres is approximately 5–100 times lower than that at similar distances in chromosome arms in several organisms [81,82,83]. Frequent crossing over at the centromere causes loss of centromeric cohesion leading to meiosis II failure and aneuploid gametogenesis [84,85]. Therefore, suppression of crossing over at centromeric regions is essential for proper chromosome segregation during meiosis II. In addition, studies in *S. pombe* have identified that Sgo1 protects Rec8 from cleavage by separase around the centromere during anaphase I [86,87,88]. Sgo1 is specifically expressed during meiosis and localizes to the centromeric region. Rec8 is phosphorylated by type I casein kinase δ/ε (CK1), which makes it susceptible to degradation by separase. In contrast, PP2A phosphatase, which interacts with Sgo1, localizes to the centromere. Therefore, at the centromere, phosphorylation of Rec8 by CK1 was reversed by PP2A. Consequently, Rec8 at the centromere is protected from degradation by separase [89,90,91]. Furthermore, when the expression of mouse shugoshin is suppressed in oocytes, Rec8 at the centromere is degraded prematurely in anaphase I, consistent with findings in *sgo1∆* mutants of *S. pombe* [92,93]. Thus, meiotic cohesin protection mechanism of Sgo1 is evolutionarily conserved. Therefore, many crucial processes are regulated by Rec8 cohesin during meiosis, which is an essential event for sexual reproduction in eukaryotes.

## 4. Roles of Meiotic Cohesin in Chromosome Architecture

Forced expression of Rad21 during meiosis in the absence of Rec8 partially recovers sister chromatid cohesion, but does not compensate for defects in meiosis-specific chromosome events, including reductional segregation [65,69]. Moreover, formation of SCs in *S. cerevisiae* and the axial structure of chromosomes in *S. pombe* during meiosis are defective in *rec8∆* mutants, even with the ectopic expression of Scc1/Rad21 [35,69]. Therefore, Rec8 cohesin may play additional roles other than cohesion in regulating meiotic chromosome behavior, such as SC formation, associated with axial structure.

### 4.1. Formation of Axial Elements

The axial element is a precursor of the lateral element of the SC, and corresponds to the LinE in in asynaptic organisms such as *S. pombe*. In the SC-forming organism *S. cerevisiae*, the formation of the axial element during the meiotic prophase is defective in *rec8* mutants, specifically affecting Hop1 and Red1, which are both structural components of the axial element and do not form linear structures in *rec8* mutants, as observed by electron microscopy of the meiotic prophase nuclei [15]. Rec8 interacts with Red1 (Rec10 in *S. pombe* and SYCP2 in mice) and Hop1 [94]. Because Rec8 is required for correct localization of Red1 and Hop1 to the chromosomal axis sites [94,95], Rec8 is essential for recruiting Red1 and Hop1 to a subset of meiotic chromosomal positions, at least in *S. cerevisiae* [94].

In *S. pombe*, Rec8 cohesin contains a meiosis-specific SA3 subunit called Rec11. No obvious homolog of SA3 has been found in *S. cerevisiae*. Importantly, it has been shown that the N-terminal region of Rec11 is phosphorylated by CK1 during meiosis [96,97]. This phosphorylation induces an interaction between Rec11 and Rec10 (a homolog of *S. cerevisiae* Red1, which is a structural component of LinE in *S. pombe*). This interaction further results in the recruitment of Rec10 and other LinE components, such as Hop1, Rec25, and Rec27, onto chromatin, and induce LinE assembly along sister chromatids [96]. As implied by its gene name, Rec8, Rec10, and Rec11 in *S. pombe* were originally isolated as mutants deficient in meiotic recombination [98]. LinE is necessary for the recruitment of recombination apparatus, such as Rec12 (Spo11 homolog in *S. pombe*), along the chromatin [99]. Therefore, the mechanism by which cohesin and LinE cooperate to regulate recombination in *S. pombe* may explain the recombination-defective phenotype in these mutants.

In mice, in addition to Rec8, the meiosis-specific subunits SA3 (Rec11 homolog), kleisin subunit Rad21L, and Smc1β are required for the assembly of axial elements during the meiotic prophase [20,21,22,23,24,25,100,101]. Moreover, chromatin-bound cohesins, including SA3, are highly phosphorylated during the meiotic prophase in mouse spermatocytes [102]. Therefore, it is possible that phosphorylation of the cohesin complex contributes to the meiotic chromosome axis assembly in mammalian meiosis, similar to *S. pombe*. Rec8 cohesin is also required for the assembly of axial elements in *Arabidopsis thaliana* and *Caenorhabditis elegans* [16,17,18,103]. Therefore, the meiosis-specific cohesin complex has conserved functions in a wide range of organisms and regulates meiotic recombination by assembling axis structures along sister chromatids by recruiting structural components of the axial element.

### 4.2. Formation of the Axis-Loop Chromatin Structure

Importantly, studies using mouse spermatocytes have demonstrated that the loss of SYCP2 or SYCP3, structural components of the axial element, disrupts the formation of axial elements, but generally results in retention of the cohesin axis, which is visualized by immunostaining of cohesin subunits in meiotic prophase cells [25,104,105]. Similarly, in *S. pombe*, LinE formation-defective mutants, but not *rec8* mutants, maintain structural properties of chromatin to withstand oscillatory nuclear movement during meiotic prophase [54,96]. These results strongly suggest that Rec8 cohesin-induced chromosome structure is independent of the axial elements (or LinEs in *S. pombe*). In addition, previous electron microscopy observations have detected axial structures with chromatin loops (axis-loop chromatin structure), such as those now thought to be mediated by Rec8 cohesin [33,34,35,106]. Furthermore, recent studies in several organisms using high-throughput chromosome conformation capture (Hi-C) have depicted the configuration of meiotic chromosomal structures [33,107,108,109,110,111,112,113,114,115]. Importantly, it has been reported that Rec8 localization correlates with regions of contacts detected by Hi-C, which represent the bases of chromatin loops in *S. cerevisiae* [33,34,116]. Moreover, these loops are lost in the absence of Rec8 cohesins [33]. Therefore, it is now clear that the axis structure with chromatin loops is mediated by Rec8 cohesin during meiosis.

In contrast, chromatin loops are undetectable via Hi-C in mammalian meiosis [107,109,110], despite clear observation of chromatin loop structures in mouse meiotic chromosomes by electron microscopy [117,118]. It has been argued that the chromosomal position of Rec8 or the loop location varies from cell to cell in mammals [107]. In somatic cells of several organisms, Rad21/Scc1 cohesins have the ability to form higher-order structures called topologically associated domains (TADs) by pulling together distant regions of chromosomes, which can at times be up to several Mbp [119,120,121,122,123]. In mammalian meiosis, TAD signals observed in mitotic interphase cells mostly disappear [107,109,110]. This suggests that switching of protein subunits is essential to construct chromosomes that are ready for subsequent meiotic recombination, probably by exchanging cohesins from mitotic RAD21 to its meiotic counterparts along the chromosomes.

Recently, Hi-C analysis in *S. pombe* has revealed the appearance of chromatin loops between known Rec8-binding sites upon entry into meiosis [35]. These loop structures are completely lost in *rec8∆* cells. However, in *rec12∆* (Spo11 homolog of *S. pombe*) mutants, in which recombination never occurs, these loop structures are normally maintained. Moreover, the normal formation of loop structures is also observed in *rec10∆* cells, in which LinE is not assembled. In addition, similar to those in mammalian spermatocytes, with the emergence of Rec8-dependent loop structures, TAD-like signals observed in mitotic interphase cells mostly disappear during the meiotic prophase [35]. This could be mainly due to the switching of kleisin subunits from Rad21 to Rec8, which occurs upon commitment to meiosis. Although Rec8-cohesin-dependent loop structures have been detected in *S. pombe* and *S. cerevisiae* [33,34,35], the mechanism through which loop formation and sister chromatid cohesion are coordinately regulated as well as the contribution of Rad21/Scc1 cohesin remains unclear. It has been proposed that two separate populations of Rec8 cohesin act on loop formation and cohesion [34]. In mouse oocyte meiosis, it has been suggested that RAD21/SCC1 and REC8 cohesins have distinct functions in chromatin loop formation and cohesion, respectively [36,108]. Functions of the kleisin subunits in meiotic chromatin structure may differ among species.

**Figure 6:**
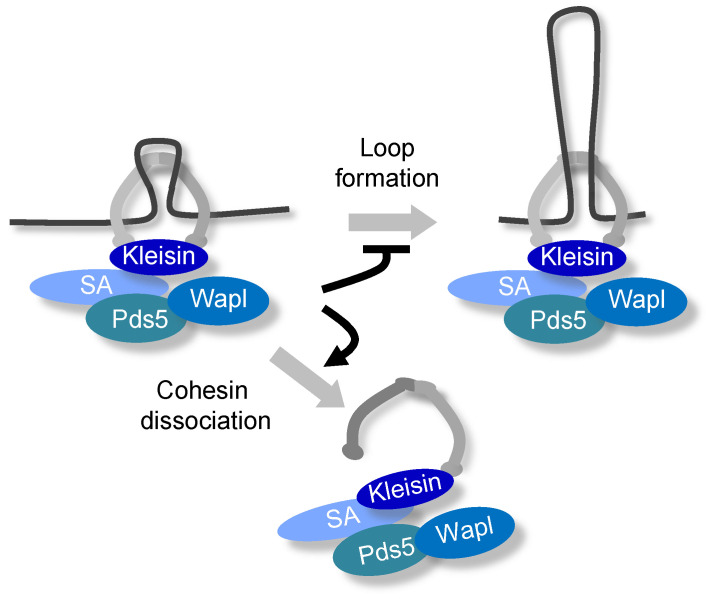
Proposed functions of Pds5/Wapl in the regulation of cohesin. PDS5/WAPL inhibits loop formation and promotes cohesin dissociation from chromatin. Conformational changes or unknown regulatory factors may be involved at each step to modulate the functions of Pds5 and Wapl.

**Figure 7:**
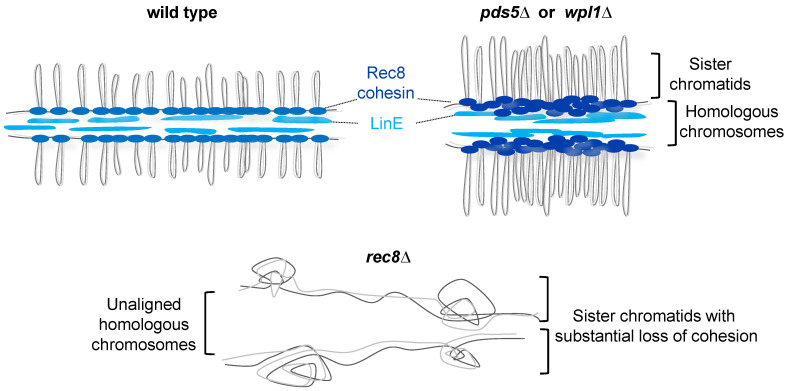
Chromosome architectures regulated by Pds5, Wpl1 and Rec8 in *S. pombe.* The Rec8-dependent loop-axis structure and linear element (LinE) are shown. It is unclear whether the same Rec8 cohesin complex is responsible for both cohesion and loop formation or whether different subsets of Rec8 cohesin are used at each step separately.

Considering the cohesin-mediated axial loop structure of chromosomes during meiosis, it is necessary to consider the cohesin regulators Pds5 and Wpl1/WAPL. Pds5 is a cohesin-associated factor thought to maintain cohesion during mitosis [124,125,126,127]. However, it has been shown that WAPL interaction with PDS5 promotes the release of mitotic cohesin from the chromosome arms prior to anaphase onset independently of separase in mammals (Figure 6) [128,129,130]. Additionally, in *S. cerevisiae*, Wpl1 releases Rec8 cohesin from chromosomes during the meiotic prophase [131,132]. Interestingly, consistent with findings in WAPL-depleted mammalian somatic cells [108,131,133,134,135,136], shortening of the chromatin axis was also commonly observed in Pds5 and Wpl1/WAPL-depleted meiotic cells in *S. pombe*, *S. cerevisiae*, *C. elegans*, and mice [35,54,131,132,136,137,138]. Concomitant with shortening of the axial structure, lengthening of the chromatin loop has also been observed [35,133,139]. Taken together, this suggests that Pds5 and Wpl1/WAPL have a conserved function that maintains the full axis length of meiotic chromosomes (Figure 6 and Figure 7). Intriguingly, in *S. cerevisiae* and *S. pombe* meiotic cells, the loss of Pds5 results in a pairing defect similar to that observed in *rec8∆* meiotic cells [55,137]. Moreover, *S. pombe* meiotic *wpl1∆* cells are defective in homologous pairing [35]. These results suggest that Pds5 and Wpl1/WAPL modulate Rec8 cohesin axis-loop structures, acting as a structural platform that facilitates pairing and subsequent recombination during meiosis.

## 5. Perspective

In this review, we highlighted Rec8 cohesin-dependent formation of the axis-loop structure of chromatin and its role in pairing and recombination during the meiotic prophase. In mammalian somatic cells, *cis*-looping of the distal chromatin or TAD formation occurs via the SCC1/RAD21 cohesin during interphase. Interestingly, the SCC1/RAD21 cohesin forms an axial structure in WAPL-depleted mammalian somatic cells. Thus, Rad21 cohesin also seems to have the potential to regulate the formation of axial structures, albeit in a mechanism different from Rec8. In mammalian somatic cells, RAD21 cohesin complexes are anchored to chromatin via the CCCTC-binding factor (CTCF) [139,140,141,142], leading to the creation of TADs. To date, it is unknown whether CTCF functions in establishing meiotic chromosomes with an axis and looping. Recently, a loop extrusion model has been proposed to create a chromatin loop via SMC protein complexes, including cohesin. In this proposed model, the SMC protein complex interacts with DNA, which reels and extrudes it through its ring structure to form a DNA loop [143,144,145,146,147]. The human RAD21/SCC1 cohesin can extrude DNA with the cohesin loading factors NIPBL and MAU2 in vitro [143], although it is unknown whether the loop extrusion model is applicable in vivo, whereas CTCF is a conserved factor only in vertebrates [148], NIPBL/Scc2 and MAU2/Scc4 are conserved in a wide range of eukaryotes from yeasts to humans. Therefore, it is possible to consider a scenario in which Rec8 cohesin might function with a cohesin loader for axes and loops along the meiotic chromosomes. 

Loss-of-function mutations of cohesin-related factors are known to be responsible for human diseases called “cohesinopathies”, such as Cornelia de Lange Syndrome which is characterized by mental retardation, facial dysmorphism, upper limb abnormalities, and growth delay [149,150]. In addition, defects in the regulation of meiotic recombination by meiotic cohesins are closely associated with miscarriages and congenital abnormalities in humans [5,6]. Therefore, it is important to elucidate the contribution of cohesin-induced axes and loop structure formation to the etiology of these human diseases.
